# COVID-19 Pandemic–Related Disruptions in Routine Cirrhosis Care and Associated Clinical Outcomes among San Francisco Bay Area Veterans

**DOI:** 10.1016/j.gastha.2025.100711

**Published:** 2025-05-27

**Authors:** Jessica B. Rubin, Rebecca Loeb, Alexander Monto, Robert J. Wong, Ramsey Cheung, Steven L. Batki, Michael J. Ostacher, Hui Shen, Katherine J. Hoggatt, Derek D. Satre, Mandana Khalili

**Affiliations:** 1 Division of Gastroenterology and Hepatology, University of California San Francisco, San Francisco, California; 2 VA Medical Center, San Francisco Veterans Affairs Healthcare System, San Francisco, California; 3 Division of Gastroenterology, University of California Los Angeles, Los Angeles, California; 4 Veterans Affairs Greater Los Angeles Healthcare System, Los Angeles, California; 5 Division of Gastroenterology and Hepatology, Stanford University School of Medicine, Palo Alto, California; 6 VA Medical Center, Veterans Affairs Palo Alto Healthcare System, Palo Alto, California; 7 Department of Psychiatry and Behavioral Sciences, Weill Institute for Neurosciences, University of California San Francisco, San Francisco, California; 8 Department of Psychiatry and Behavioral Sciences, Stanford University School of Medicine, Palo Alto, California; 9 Center for Data to Discovery and Delivery Innovation (3DI), San Francisco Veterans Affairs Health System, San Francisco, California; 10 Department of Medicine, University of California San Francisco, San Francisco, California; 11 Division of Research, Kaiser Permanente Northern California, Oakland, California; 12 Division of Gastroenterology and Hepatology, Zuckerberg San Francisco General Hospital, San Francisco, California

**Keywords:** Health Disparity, SARS-CoV-2, Telemedicine, Alcohol-Associated Liver Disease, Quality of Care

## Abstract

**Background and Aims:**

The COVID-19 pandemic disrupted health-care delivery for chronic conditions managed in specialty care and particularly among socioeconomically disadvantaged and Veteran populations. Clinical outcomes in cirrhosis are highly dependent on routine care, including clinic visits, labs, and imaging. This study examines the pandemic's impact on adherence to cirrhosis-related quality indicators and clinical outcomes among Bay Area Veterans receiving hepatology care.

**Methods:**

We conducted a retrospective cohort study of Veterans with cirrhosis seen at San Francisco and Palo Alto Veterans Affairs hepatology clinics (March 2017–March 2023). Adherence to quality indicators—hepatocellular carcinoma (HCC) screening, lab monitoring, and hepatology visits—was compared between prepandemic (2017–2020) and pandemic-postpandemic (2020–2023) periods. Multivariable logistic regression assessed the effect of time and pandemic phase on clinical outcomes, including new hepatic decompensation, new HCC, and liver-related hospitalizations or death.

**Results:**

Among 1501 Veterans with cirrhosis (median age 67 years, 97% male), adherence to quality indicators declined during the pandemic, with hepatology visits decreasing from 1.5/year to 1.0/year, and telehealth use increasing sevenfold. Overall mortality rates were higher during the pandemic (21% vs 17%) possibly related to nonliver causes, since on multivariable models, the pandemic phase was associated with lower odds of new decompensation (adjusted odds ratio (aOR) 0.87, 95% confidence interval (CI) 0.5–0.9, *P* = .01) and HCC diagnosis (aOR 0.7, 95% CI 0.5–0.95, *P* = .02); liver-related hospitalizations or death also declined over (aOR 0.98, 95% CI 0.97–0.99, *P* < .001).

**Conclusion:**

The pandemic significantly disrupted cirrhosis care among Bay Area Veterans, potentially resulting in “health debt” that may affect outcomes in coming years. Targeted interventions should re-engage patients, addressing these care gaps and improving clinical outcomes.

## Introduction

The prevalence of cirrhosis has risen exponentially over the past decade, as has cirrhosis-related morbidity and mortality.^1^, ^2^, ^3^ Regular disease monitoring, screening, and prevention of complications—including ascites, gastrointestinal bleeding, encephalopathy, and hepatocellular carcinoma (HCC)—are essential for improving patient outcomes and reducing costs.^4^^,^^5^ The COVID-19 pandemic profoundly affected health-care delivery across most chronic diseases, including cirrhosis, due to delays in this essential routine care.^6^^,^^7^

The San Francisco Bay Area experienced one of the lowest COVID-19 mortality rates across large United States metropolitan areas, achieved through longer and more restrictive stay-at-home orders.^8^, ^9^, ^10^ These policies may have slaalso led to more disruptions in health-care than in other regions, particularly for vulnerable populations with limited access to telehealth and prescriptions, and with higher rates of COVID-19 infections.^11^^,^^12^

Despite better COVID-19 outcomes, the Bay Area has significantly *worse* cirrhosis-related outcomes than other metropolitan areas, with higher rates of cirrhosis and rising cirrhosis-related mortality.^3^^,^^13^^,^^14^ The region's socioeconomic vulnerability—including racial/ethnic diversity, income inequality, and high rates of alcohol and other substance use^12^^,^^15^—increase the risk of developing cirrhosis and adverse outcomes.^16^^,^^17^ But how the pandemic and restrictive COVID-19 policies specifically affected Bay Area cirrhosis patients—and Veteran populations in particular—remains largely unknown. Additionally, whether the subsequent easing of restrictions in mid-2022 altered the trajectory of cirrhosis-related care and outcomes is unknown.

We hypothesize that the pandemic significantly impacted cirrhosis management and outcomes in the Bay Area, with incomplete recovery despite the easing of restrictions. To test this hypothesis, we utilized a longitudinal cohort of Bay Area Veterans with cirrhosis receiving hepatology care to compare rates of adherence to cirrhosis-related quality indicators (lab monitoring, hepatology visits, and HCC screening), alcohol use and substance use patterns, and associated clinical outcomes in the prepandemic and pandemic and postpandemic periods.

## Methods

### Cohort Definition

We conducted a retrospective cohort study using data from the Veterans Affairs (VA) Corporate Data Warehouse linked with Center for Medicare/Medicaid Services (CMS) data. Veterans aged 18+ with cirrhosis seen in either the San Francisco VA (SFVA) or the Palo Alto VA (PAVA) hepatology clinics between March 1, 2017, and February 28, 2022, were included. Both sites are tertiary referral centers affiliated with academic medical centers in the San Francisco Bay Area. Patients were required to be “active” within the VA system during the year preceding their first hepatology clinic visit, defined as having at least one VA/CMS clinical encounter or prescriptions fill. Cirrhosis diagnosis required at least one validated International Classification of Diseases, 10th Revision (ICD-10) code for cirrhosis (see Table A1), within the 2 years before or 3 months following the first hepatology visit.^18^ Cohort entry was defined by the first hepatology clinic visit *within the study period* (though may have been a new or follow-up visit). Patients were followed until death or until March 1, 2023, ensuring at least 1 year of follow-up.

### Demographic, Comorbidity, and Cirrhosis Variables

Extracted data included demographics, ICD-10 codes for inpatient and outpatient encounters (at VA or paid for by VA/CMS), hepatology clinic visit information (generated using stop code 337),^19^ and laboratory values. Demographics at cohort entry included age, gender, race and ethnicity (combined to race/ethnicity variable), marital status, urban/rural status, and national-level area deprivation index. VA health factor data determined smoking status. The Alcohol Use Disorders Identification Test—Consumption (AUDIT-C) defines hazardous drinking (≥3 for women and ≥4 for men).^20^^,^^21^ Validated ICD-10 codes defined socioeconomic hardship (Z59-60), alcohol and other substance use disorders, and common medical/psychiatric comorbidities.

Cirrhosis etiology and complications were defined using validated of ICD-10 codes (Table A1).^18^^,^^22^, ^23^, ^24^ Patients were defined as decompensated if they had at least 1 ICD-10 code for ascites (or an ascites-related complication), bleeding varices, or hepatic encephalopathy in the prior year. Cirrhosis severity was assessed by model for end-stage liver disease-sodium (MELD-Na) score closest to cohort entry.

The following variables were measured as time-varying covariates, including substance use (given the potential for changes in substance use during the pandemic phases),^25^ cirrhosis complications, and MELD-Na scores. We also collected time-varying data on COVID infections available in the Corporate Data Warehouse database, and COVID vaccinations from state registry data.

### Quality Indicators

Three measures of adherence to quality indicators included (1) HCC screening, defined as multiphase abdominal imaging or ultrasound every 6 months; (2) Lab monitoring, defined as complete MELD-Na labs (ie, on the same day) every 6 months; and (3) Hepatology clinic visits, defined by Hepatology stop code (337). While ideal lab and imaging frequency is every 6 months (ie, twice per year) based on current guidelines,^26^, ^27^, ^28^ screening once annually provides some benefit. Thus, analyses also examined the proportion of patients who had at least 1 set of labs or imaging per year. We excluded variceal screening due to changing national guidance regarding the need for endoscopy vs beta-blockers during the study period.^29^

### Clinical Outcomes

The primary study outcome was a composite outcome consisting of death or liver-related hospitalization, defined as a hospitalization with a primary diagnosis of cirrhosis or a cirrhosis complication (as defined in Table A1). Death data incorporated state registry information. Secondary outcomes included new hepatic decompensation (among previously compensated patients) and new HCC diagnosis.

### COVID-Related Time Periods

Data were aggregated into up to 6 1-year time periods per patient, starting on March 1. These years were categorized into 2 phases: the prepandemic phase (January 3, 2017, to February 29, 2020) and the pandemic/postpandemic phase (ie “pandemic phase”) (January 3, 2020, to February 28, 2023). Data from 2017 were excluded from quality indicator analyses, as most patients enrolled during 2017 and did not have complete data for that year.

### Statistical Analysis

Descriptive analyses summarized the demographic and clinical characteristics by site (SFVA and PAVA), as well as clinical characteristics, quality indicators, and outcomes by phase and by year. Differences were tested using chi-square tests (categorical variables), and Wilcoxon rank-sum tests (continuous variables). Patients were included in analyses if they entered the study cohort before or during the respective phase and were alive at the phase's start. For new HCC diagnosis or decompensation, only patients without a prior diagnosis at the phase/year start were eligible.

We conducted pooled logistic regression to test for effects of time, pandemic phase, and their interaction outcomes. Data were pooled into 2-month time periods, and censored after the event occurrence. Models adjusted for demographic and clinical factors defined a priori as most likely to impact outcomes. For these models, cirrhosis etiology was defined using ICD-based definitions as shown in Table A1. Only best-fit models were reported.

Analyses were conducted using R (v4.4.1; dR Core Team, 2024) and RStudio (v2024.09.0; Posit Software, 2024).

## Results

### Baseline Demographics and Clinical Characteristics

**Figure 1:**
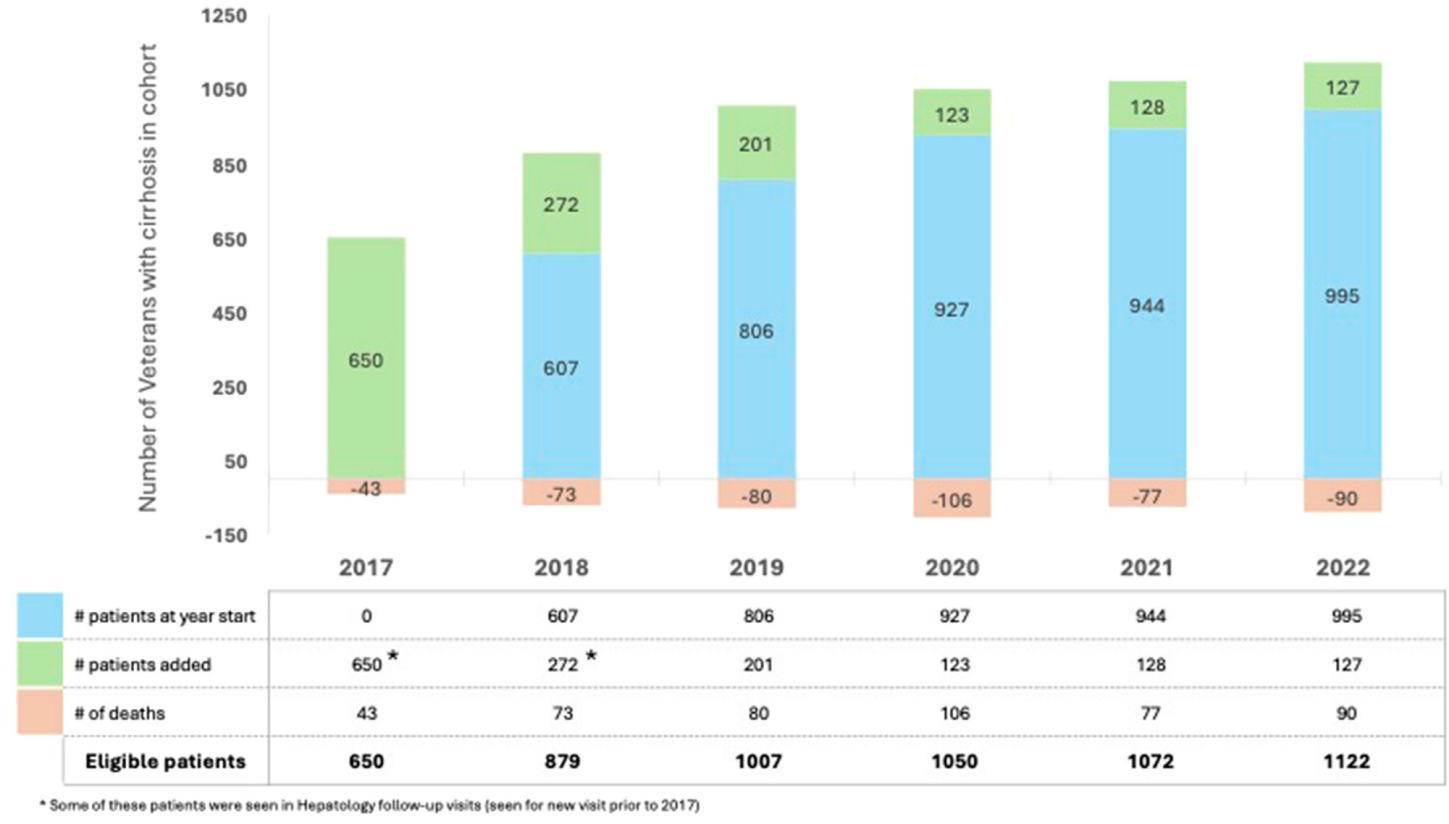
Patients entering and leaving chort, by year.

The study cohort included 1501 patients with cirrhosis seen at least once in the PAVA (n = 821) or SFVA (n = 680) hepatology clinics. Changes in cohort by year are shown in Figure 1. The number of new patients joining the cohort was significantly lower in the pandemic (mean of 126/year) than in the prepandemic period (mean 237/year). Nearly one-third of the cohort died during the study period (n = 469), with most (58%) dying during the pandemic period.

Baseline demographics in the year before study entry are shown in Table 1. The median age at study entry was 67 years. The cohort was 97% male and 59% non-Hispanic White. Nearly one-third of our cohort were current smokers and 23% had a positive AUDIT-C screen, while 43% had an ICD code for alcohol use disorder and 33% for other substance use. Medical and psychiatric comorbidities were common: 67% had hypertension, 38% had diabetes, and 29% had depression. The most common liver disease etiology was hepatitis C followed by alcohol. Median MELD-Na at the time of cohort entry was 10 (interquartile range 8–15); 24% were decompensated. A comparison of the baseline demographics and clinical characteristics between the 2 VA sites is shown in Table 1.

**Table 1:** Baseline Demographics and Clinical Characteristics of Veteran Cohorts With Cirrhosis

Characteristics	Full cohort (N = 1501)	Location of index visit		*P* value
		Palo Alto (n = 821)	San Francisco (n = 680)	
Demographics^a^				
Age, Median (IQR)	67 (62–71)	67 (62–71)	68 (63–72)	.02
Female	45 (3)	24 (3)	21 (3.1)	.9
Race/Ethnicity				<.001
Asian/Pacific Islander	58 (4)	30 (4)	28 (4)	
Black	171 (11)	81 (10)	90 (13)	
Hispanic	221 (15)	169 (21)	52 (8)	
White	882 (59)	476 (58)	406 (60)	
Other	169 (11)	65 (8)	104 (15)	
Married (current) (n = 1464)	507 (34)	313 (39)	194 (29)	<.001
Urban (n = 1500)	1160 (77)	672 (82)	488 (72)	<.001
ADI, Median (IQR) (n = 1451)	13 (4–31)	14.5 (5–34)	11 (4–27)	<.001
Socioeconomic hardship	289 (19)	134 (16)	155 (23)	.002
Substance use^a^				
Current smoker (n = 1494)	453 (30)	218 (27)	235 (35)	.002
Positive AUDIT-C (n = 995)	233 (23)	135 (24)	98 (23)	.6
Alcohol use disorder	641 (43)	332 (40)	309 (45)	.051
Substance use disorder	502 (33)	233 (28)	269 (40)	<.001
Medical and psychiatric comorbidities^a^				
Anxiety	220 (15)	121 (15)	99 (15)	.9
Depression	434 (29)	227 (28)	207 (30)	.2
PTSD	389 (26)	227 (28)	162 (24)	.1
Cancer, excluding HCC	136 (9)	69 (8)	67 (10)	.3
Chronic kidney disease	206 (14)	101 (12)	105 (15)	.1
Diabetes	565 (38)	327 (40)	238 (35)	.054
Cardiovascular disease	346 (23)	179 (22)	167 (25)	.2
Hypertension	1005 (67)	567 (69)	438 (64)	.1
Cirrhosis etiology^a^				
Hepatitis B	155 (10)	69 (8)	86 (13)	.01
Hepatitis C	876 (58)	453 (55)	423 (62)	.01
Alcohol	832 (55)	451 (55)	381 (56)	.7
MASLD	489 (33)	252 (31)	237 (35)	.1
Other	90 (6)	60 (7)	30 (4)	.02
Cirrhosis severity and complications^a^				
MELD-Na, Median (IQR) (n = 1097)	10 (8–15)	10 (8–15)	10 (7.5–14)	.06
Decompensated	361 (24)	197 (24)	164 (24)	.96
Varices				
Nonbleeding	189 (13)	99 (12)	90 (13)	.1
Bleeding	73 (5)	33 (4)	40 (6)	.1
Ascites	295 (20)	161 (20)	134 (20)	1.0
Hepatic encephalopathy	93 (6)	45 (6)	48 (7)	.2

ADI, Area Deprivation Index; IQR, interquartile range; MASLD, metabolic-associated steatotic liver disease; PTSD, post-traumatic stress disorder.

a All values represent number (percent) unless otherwise specified.

### Comparison of Patient Characteristics from the Prepandemic Phase to the Pandemic Phase

Smoking rates decreased and problematic alcohol use increased during the pandemic compared to the prepandemic period, though this was not statistically significant (Table 2). Fewer patients were missing substance use assessments during the pandemic compared to the prepandemic period. MELD-Na scores were similar during the phases, as were rates of cirrhosis-related complications. Approximately 16% of our cohort had a documented COVID-19 infection, and 58% received at least 1 COVID vaccine.

**Table 2:** Substance Use, Cirrhosis Complications, COVID Infections/Vaccinations, Quality Metrics, and Outcomes by Pandemic Phase

All values are number (%) unless otherwise stated	Prepandemic	Pandemic/Post	*P* value
Included dates	January 3, 2017, to February 29, 2020	January 3, 2020, to February 28, 2023	
Number of 1-y time periods	3	3	-
Patients in cohort^a^ (n)	1123	1305	-
Substance use			
Smoking status			
Current smoker	317 (37)	353 (32)	
Nonsmoker	551 (63)	764 (68)	
Missing	255	188	.02
Positive AUDIT-C screen	162 (18)	227 (20)	
Negative	755 (82)	887 (80)	
Missing	206	191	.1
Alcohol use disorder	537 (48)	670 (51)	.1
Cirrhosis severity and complications			
MELD-Na, Median (IQR)	10 (8–14)	10 (8–15)	.1
Decompensated	457 (41)	535 (41)	.9
Varices			
Nonbleeding	271 (24)	279 (21)	.1
Bleeding	74 (7)	104 (8)	.2
Ascites	299 (27)	357 (27)	.7
Hepatic encephalopathy	142 (13)	178 (14)	.5
COVID-related statistics			
COVID infection	-	209 (16)	-
First COVID-19 vaccination	-	759 (58)	-
Quality metrics			
Number of clinic visits attended	2296	1937	-
Percent of visits conducted by telehealth	11%	61%	<.001
Average number of visits/year per patient	1.5	1.0	<.001
ALT monitoring^b^			
≥1 in every year	748 (67)	828 (63)	-
≥2 in every year	502 (45)	459 (35)	-
MELD-Na monitoring^b^			
≥1 in every year	504 (45)	440 (34)	-
≥2 in every year	272 (24)	206 (16)	-
Hepatocellular carcinoma screening^b^			
≥1 in every year	686 (61)	705 (54)	-
≥2 in every year	402 (36)	248 (19)	-
Outcomes			
≥1 liver-related admission	180 (16)	182 (14)	.2
Death	196 (17)	273 (21)	.03
New decompensation^c^	99 (15)	100 (13)	.6
New hepatocellular carcinoma diagnosis^c^	73 (7)	71 (6)	.3

ALT, alanine aminotransferase; IQR, interquartile range.

a Patients alive at start of phase, who entered study during the phase or earlier.

b Eligibility required entering the cohort before March 1st of the study year, data only included for study years 2018 or later. *P* values were not calculated, as phases were not equal in length (2 years vs 3 years).

c Only eligible if no prior decompensation or hepatocellular carcinoma diagnosis.

### Cirrhosis-Related Quality Metrics

**Figure 2:**
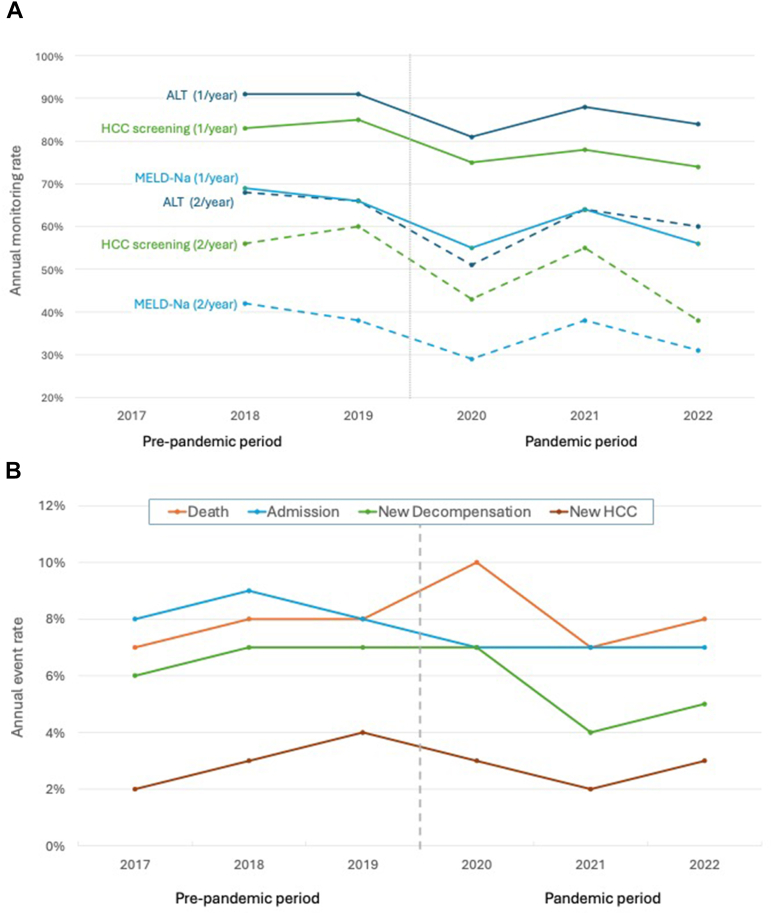
(A) Annual rates of labs and HCC screening, 2018–2022. (B) Annual rates of clinical outcomes, 2017–2022. ALT, alanine aminotransferase.

There were 2296 hepatology clinic visits during the prepandemic phase and 1937 visits during the pandemic phase (Table 2). The number of annual visits to hepatology clinics decreased during the pandemic phase (1.5/year to 1.0/year, *P* < .001), and many more were completed by telehealth (ie telephone or video visit) (11% vs 61%, *P* < .001). While two-thirds of our cohort got at least one alanine aminotransferase checked per year in 2018 and 2019, significantly fewer had regular MELD-Na labs during the pandemic period. Similarly, while 61% of our cohort received at least 1 HCC screening/year in the prepandemic period, rates dropped to 54% postpandemic, with even lower rates of twice-yearly imaging studies. Table 3 shows annual rates of visits, labs, and imaging. As shown in Figure 2A, 2020 had the lowest rates of both lab and imaging monitoring. Rates sharply increased in 2021 then subsequently decreased again in 2022.

**Table 3:** COVID and Association With Quality Metrics and Outcomes by the Annual Period, 2017–2022

Outcome variables	Prepandemic			Pandemic period		
	2017	2018	2019	2020	2021	2022
Eligible patients^a^	650	879	1007	1050	1072	1122
COVID						
COVID-19 infection	-	-	-	33 (3)	89 (8)	87 (8)
COVID-19 vaccination	-	-	-	452 (43)	303 (28)	4 (0)
Quality metrics						
Clinic visit attended	503	826	967	500	618	819
Average visits/y	1.6	1.5	1.6	0.9	1.0	1.2
% Telehealth visits	11%	11%	10%	74%	53%	58%
ALT monitoring^b^	-					
≥1 in every year		555 (91)	730 (91)	753 (81)	826 (88)	840 (84)
≥2 in every year		413 (68)	528 (66)	472 (51)	607 (64)	604 (60)
MELD-Na monitoring^b^	-					
≥1 in every year		420 (69)	532 (66)	512 (55)	602 (64)	560 (56)
≥2 in every year		253 (42)	307 (38)	268 (29)	358 (38)	312 (31)
HCC screening^b^	-					
≥1 in every year		504 (83)	686 (85)	695 (75)	740 (78)	742 (74)
≥2 in every year		338 (56)	480 (60)	403 (43)	515 (55)	381 (38)
Outcomes						
Death	43 (7)	73 (8)	80 (8)	106 (10)	77 (7)	90 (8)
≥1 admission	51 (8)	81 (9)	85 (8)	76 (7)	78 (7)	75 (7)
New Decompensation^c^	22 (6)	37 (7)	40 (7)	42 (7)	24 (4)	34 (5)
New HCC^c^	13 (2)	22 (3)	38 (4)	25 (3)	17 (2)	29 (3)

ALT, alanine aminotransferase.

a Patients alive on March 1 of the year, who entered the study during the study year or earlier.

b Eligibility required entering the cohort before March 1 of the study year; data only included for study years 2018 or later to ensure adequate follow-up.

c Only eligible if no prior decompensation or hepatocellular carcinoma diagnosis.

### Cirrhosis-Related Clinical Outcomes

We found that 23% of our cohort developed new decompensation during the study period, with annual rates ranging from 4% to 7%. Documented rates of decompensation were higher in prepandemic compared with pandemic time periods (15% vs 13%), though this was not statistically significant (*P* = .6) (Table 2). Additionally, 11% of our cohort was diagnosed with HCC during the study period, though rates were similar between the time periods (7% prepandemic vs 6% pandemic, *P* = .3); and 16% had a liver-related hospitalization in the prepandemic compared with 14% in the postpandemic phase (*P* = .2). Despite this, overall mortality rates were higher during the pandemic phase (21%) compared with prepandemic phase (17%) (*P* = .03). Annual rates of our clinical outcomes are shown in Table 3 and Figure 2B.

The results of our best-fit multivariable regression models for each of our clinical outcomes are shown in Table 4. We found that liver-related admissions or deaths *decreased* over time on average, controlling for clinical and demographic variables (adjusted odds ratio (aOR) 0.98, 95% confidence interval (CI) 0.97–0.99, *P* < .001). Risk factors for *increased* risk of this composite outcome included: those with older age, socioeconomic hardship, decompensation at baseline, and alcohol etiology (see aORs in Table 4). Similarly, adjusted odds of new decompensation were lower during the pandemic phase compared to the prepandemic phase (aOR = 0.7, 95% CI 0.5–0.9, *P* = .01), as were the odds of new HCC diagnosis (aOR = 0.7, 95% CI 0.5–0.95, *P* = .02).

**Table 4:** Best Fit Pooled Logistic Regression Models for the Effect of Time (in 2-Month Increments) And/Or Pandemic Phase on Primary and Secondary Clinical Outcomes^a^

Variables	Liver-related admission or death		New decompensation		New HCC	
	aOR (95% CI)	*P* value	aOR (95% CI)	*P* value	aOR (95% CI)	*P* value
Time	**0.98 (0.97–0.99)**	**<.001**^b^	-	-	-	
Pandemic phase	-	-	**0.7 (0.5–0.9)**	**.01**^b^	**0.7 (0.5–0.95)**	**.02**^b^
Age	1.02 (1.01–1.03)	<.001^b^	1.03 (1.00–1.05)	.02^b^	1.01 (0.98–1.03)	.5
Socioeconomic hardship	1.5 (1.2–1.9)	<.001^b^	1.3 (0.9–1.9)	.14	0.7 (0.4–1.1)	.2
Decompensation	2.5 (2.0–3.1)	<.001^b^	-	-	0.7 (0.4–1.1)	.1
Alcohol etiology	1.9 (1.6–2.4)	<.001^b^	3.1 (2.3–4.2)	<.001^b^	1.5 (1.1–2.3)	.03^b^

Bold variables are primary predictors for each model.

CI, confidence interval.

a All models were adjusted for age, socioeconomic hardship, liver disease etiology, gender, race, rural location, and site of first visit, as well as interaction between time and pandemic phase. For admission/death and new HCC, models, we also controlled for baseline decompensation. Only covariates with ≥1 significant association are shown in table.

b Significant at *P* < .05 level.

## Discussion

This study provides insight into the impact of the COVID-19 pandemic on cirrhosis-related care and outcomes among Veterans in the San Francisco Bay Area, informing our understanding of care disruptions in cirrhosis more broadly. We found decreased adherence to key cirrhosis-related quality metrics during the pandemic, including hepatology clinic visits, laboratory monitoring, and HCC screening, along with increased reliance on telehealth, which has persisted through March 2023. Additionally, mortality rates were higher in the pandemic period, potentially due to non–liver-related causes, as rates of new decompensation, new HCC diagnoses, and liver-related admissions decreased during the pandemic period.

Disruptions in preventive care have been widely documented during the early pandemic years,^30^, ^31^, ^32^, ^33^ but their long-term impact, often referred to as “health debt”, remains unclear.^34^ Few studies have evaluated health debt among cirrhosis patients—in whom routine clinic, lab, and imaging are essential. While a national VA study reported reduced HCC and variceal screening in cirrhosis patients during the pandemic,^35^ our study builds on this prior work by evaluating a more comprehensive set of quality metrics over a longer time period, and focusing on a region with significant COVID-related restrictions. In addition to a decline in HCC screening, which has been noted previously,^35^, ^36^, ^37^ we found a decline in other hepatology clinic visits and lab monitoring as well, even when using more liberal definitions of adherence. Additionally, fewer new cirrhosis patients were seen in study clinics during the pandemic likely due to reduced cirrhosis detection or delayed referral. Despite some recovery in visits and monitoring in later years, new hepatology referrals remained at almost half of prepandemic levels by the end of our study period.

A notable shift in care delivery was the sharp rise in telehealth visits, which increased over sevenfold from 2019 to 2020 and remained elevated. While telehealth improved care access during restrictions, it may not have ensured adherence to in-person care such as labs and imaging, potentially exacerbating disparities among patients with limited access to technology. The Bay Area's stringent COVID-19 policies may further restricted health-care access for vulnerable populations. Prior work highlights that this persistent reliance on telehealth to provide hepatology care may particularly impact care for certain demographics, such as Hispanic ethnicity, older adults, and those with alcohol use disorder.^38^^,^^39^ Given high rates of these characteristics among cirrhosis patients, it is critical to balance telehealth's advantages with the necessity of in-person evaluations.

Despite declines in quality metric adherence, we did not observe an increase in new hepatic decompensation or liver-related hospitalizations; in fact, multivariable models suggested declines in these outcomes. These findings align with prior studies^40^ and may reflect underdiagnosis due to reduced health-care utilization or patients deferring care until complications become severe. We recognize that, aside from HCC screening, there is limited evidence guiding optimal intervals for lab monitoring and clinic visits for patients with cirrhosis. Therefore, our findings raise a hypothesis—worthy of further investigation—that some subgroups of cirrhosis patients may not require routine MELD-based lab testing or clinic visits every 6 months. Finally, the absence of increased liver-related admissions in our study suggests that the higher mortality observed during the pandemic may have been driven primarily by nonliver causes, such as COVID-19 itself, particularly given the vulnerability of this population to infection-related complications. Unfortunately, we lack cause-of-death data to confirm this.

We also found a slight but significant decrease in new HCC diagnoses, consistent with prior studies that focused on the early years of the pandemic.^35^^,^^36^^,^^41^^,^^42^ This study builds on this prior work, finding that even as late as March 2023, rates of HCC detection remained lower than prepandemic rates. Given lower HCC screening rates during the pandemic, a “catch-up” period with more advanced diagnoses may occur in the coming years. Efforts should focus on reengaging patients who experienced screening disruptions to detect HCC at earlier stages.

Exploratory analyses examined whether differences in cirrhosis-related complications were related to changes in alcohol and other substance use disorders, particularly in our population, which has high baseline rates of substance use and socioeconomic disadvantage. We found a decrease in documented tobacco use during the pandemic, and a slight nonsignificant increase in alcohol use. Overall, studies have largely found an increase in rates of alcohol use and high-risk alcohol use among both liver disease patients and the general population during the pandemic.^25^^,^^43^^,^^44^ If there was, in fact, an increase in alcohol use, it was not captured in this cohort, but may result in rising cirrhosis diagnoses and complications in coming years.

This study has some limitations. First, we only captured care provided by or paid for by the VA, potentially excluding services accessed elsewhere. Second, reliance on ICD-10 codes may have underestimated cirrhosis complications during the pandemic, particularly given the decline in health-care utilization. Third, findings may not be generalizable to non-VA populations or to regions with different COVID-19 policies and health-care infrastructure, but we do believe they highlight important lessons regarding the impact of any future policy changes (even nonpandemic changes) on cirrhosis care delivery and outcomes. Fourth, while we identified pandemic-related changes in care, we likely did not capture the full impact on clinical outcomes due to the relatively short follow-up period. Moreover, we chose to focus on patients seen in hepatology clinics; disruptions may have been even more pronounced among those managed solely by primary care.

## Conclusion

This study deepens our understanding of how the COVID-19 pandemic disrupted cirrhosis care and affected clinical outcomes. Unlike prior work, we focused on a Veteran population with high social vulnerability living in a region with some of the nation's most restrictive COVID-19 policies. We assessed a broader range of quality metrics and extended our analysis longer, through March 2023. Our findings reveal persistent gaps in cirrhosis care in the wake of the COVID-19 pandemic that remain more than 2 years after the easing of COVID-era restrictions. These results carry critical implications for the VA and other health-care systems, particularly those in regions like the Bay Area with stringent public health measures, which must now prepare for a prolonged “catch-up” period. In the coming 5–10 years, providers are likely to face an influx of patients re-entering care with newly diagnosed cirrhosis or advanced complications.

Beyond immediate care deficits, our findings point to a more fundamental shift in hepatology care delivery. In this emerging “new normal”—marked by less frequent in-person visits—there is an urgent need to improve adherence to quality metrics. Efforts must especially focus on patients at highest risk of being left behind by developing specific engagement interventions targeting those with limited access to telehealth, such as older adults, individuals facing socioeconomic hardship, and patients with alcohol-associated liver disease. Further research is needed to quantify the pandemic-induced “health debt,” to clarify evolving care patterns, and to design targeted interventions that restore and enhance routine cirrhosis care.

## Supplementary Material

Table A1
