# Supplementary material for: COVID-19 Pandemic–Related Disruptions in Routine Cirrhosis Care and Associated Clinical Outcomes among San Francisco Bay Area Veterans
Source: Gastro Hep Adv. 2025 May 27;4(9):100711. doi: 10.1016/j.gastha.2025.100711 (PMC12329098; doi:10.1016/j.gastha.2025.100711)
Supplement: Table A1 [file mmc1.docx]

**Supplemental Table 1. International Classification of Diseases, 10^th^ Revision (ICD-10) Diagnosis Codes used to define cirrhosis, cirrhosis-related complications, and cirrhosis etiologies**

| Diagnosis | ICD-10 |
| --- | --- |
| Cirrhosis | K70.30  K74.69  K74.60  E83.110  K71.7  K70.31 |
| CIRRHOSIS COMPLICATIONS |  |
| Nonbleeding varices | I85.00  I86.40  I85.10 |
| Bleeding varices | I85.01  I86.41  I85.11 |
| Ascites | K70.31  K70.11  K71.51  R18.8 |
| Spontaneous bacterial peritonitis | K65.2 |
| Hepatic encephalopathy | K70.41  K71.11  K72.01  K72.11  K72.91  B19.0  B19.11  B19.21  G93.40  K72.11 |
| Hepatorenal syndrome | K76.7 |
| Hepatopulmonary syndrome | K76.81 |
| Hepatocellular carcinoma | C22.0 |
| CIRRHOSIS ETIOLOGY |  |
| Hepatitis B | B18.0  B18.1  B19.10  B19.11 |
| Hepatitis C | B18.2  B19.20  B19.21 |
| Alcohol | K70.0-9  F10.10-99 |
| Metabolic-Associated Steatotic Liver Disease (MASLD) | K75.81  K76.0  K76.9  K74.6 |
